# Cranial osteomyelitis in a patient with KID syndrome: Importance of thorough investigation in chronic wounds

**DOI:** 10.1111/ddg.15972

**Published:** 2025-10-16

**Authors:** Michael Wolfgang Höner, Anabelle Kainz, Cornelia Erfurt‐Berge

**Affiliations:** ^1^ Department of Dermatology Uniklinikum Erlangen Friedrich‐Alexander‐Universität Erlangen‐Nürnberg Erlangen Germany

**Keywords:** Keratitis ichthyosis and deafness (KID) syndrome, skin, staphylococcal skin infections, wound healing, wound management

Dear Editor,

Keratitis‐Ichthyosis‐Deafness syndrome (KID) is a rare genodermatosis with an estimated prevalence of less than 1 in 1,000,000.^1^ It is primarily caused by sporadic mutations in the *GJB2* gene, which encodes connexin 26, a protein critical for cell‐to‐cell communication.^2^ The syndrome is characterized by a triad of symptoms: keratitis, ichthyosiform desquamation (erythrokeratoderma), and neurosensory deafness. Additional features include hyperkeratosis of the palms, alopecia, and a predisposition to severe skin infections and malignancies.^3^ We present a unique case of KID complicated by cranial osteomyelitis, a previously unreported association. In 2023, a 17‐year‐old female patient with a known diagnosis of KID presented to our dermatological wound center with refractory wounds on the capillitium. The patient exhibited classic features of KID, including erythrokeratoderma, hyperkeratosis, alopecia, and neurosensory deafness. Genetical testing after birth confirmed the KID. Notably, she also presented with cachexia (BMI 13.7), extensive occipital and temporal ulcerations with hypergranulation, and frontal and parietal abscesses (Figure 1a–f). The patient's father had been managing her wound dressings at home, and she routinely wore a wig to conceal the alopecia and wounds. A cranial magnetic resonance imaging (cMRI) scan revealed chronic osteomyelitis of the skull bone corresponding to an active abscess within the frontal scalp, a finding not previously documented in KID (Figure 1g). Microbiological testing of the abscess confirmed infection with *Staphylococcus aureus*, *Staphylococcus lugdunensis*, and *Finegoldia magna*. The patient was admitted for intensive management, including an initial 6‐week course of oral antibiotic treatment with cotrimoxazole and metronidazole. Surgical debridement and abscess evacuation were performed multiple times, with histological examination ruling out malignant transformation. For the duration of antibiotic therapy, no new abscesses formed. Clinically, however, recurrent abscess formation occurred after discontinuation of antibiotic therapy, particularly over previously infected cranial areas (frontal, occipital, and parietal), suggesting persistent inflammatory activity. Neurosurgical intervention was discussed but considered unfavorable due to the patient's impaired wound healing and was also declined by the patient herself. Furthermore, she refused a prolonged inpatient stay for intravenous antibiotic therapy, which limited treatment options for the chronic osteomyelitis. Wound care involved daily dressing changes with polyhexanide solution, siliconized wound gauze for areas with more erosive lesions, and absorbent dressings in areas with more exudation. Dressing changes had to be administered initially under analgesia with piritramide. Post‐discharge, professional wound management and home care were continued. Surgical debridement significantly reduced pain and improved cervical mobility, which had been restricted by scar tissue reaching up to the jugulum. Prolonged occlusion and friction caused by the daily use of a wig may have contributed to local irritation and infection; alternatives such as headscarves were declined by the patient for psychosocial reasons. The pronounced cachexia was interpreted as a consequence of the chronic infection. Laboratory testing at presentation showed elevated ferritin (acute‐phase response), reduced vitamin D and folate levels, normal vitamin B12 levels; vitamin C and zinc were not determined. To address the erythrokeratoderma and hyperkeratosis, systemic therapy with acitretin, a retinoid, was initiated at 0.3 mg/kg body weight (BW) and increased to 0.6 mg/kg BW over time. Given the patient's young age, the potential adverse effects of long‐term systemic retinoid therapy – including mucocutaneous dryness, skeletal changes, and teratogenicity – were carefully discussed. Regular laboratory and clinical monitoring were initiated to ensure safe use. This treatment led to a marked reduction of the erythrokeratoderma as previously described.^4^ Furthermore, a high‐caloric and protein‐rich liquid nutrition was added. These interventions collectively resulted in substantial improvement in the patient's wound healing, overall clinical condition, and quality of life. Despite the known impairment of wound healing in KID syndrome, thorough diagnostic evaluation is essential to detect additional, potentially serious causes of chronic wounds, as demonstrated in this case.^5^ Preventive strategies in patients with KID syndrome include regular clinical monitoring, early treatment of minor skin lesions, avoidance of occlusive scalp coverings, and patient education to minimize the risk of severe cutaneous infections.

**FIGURE 1:**
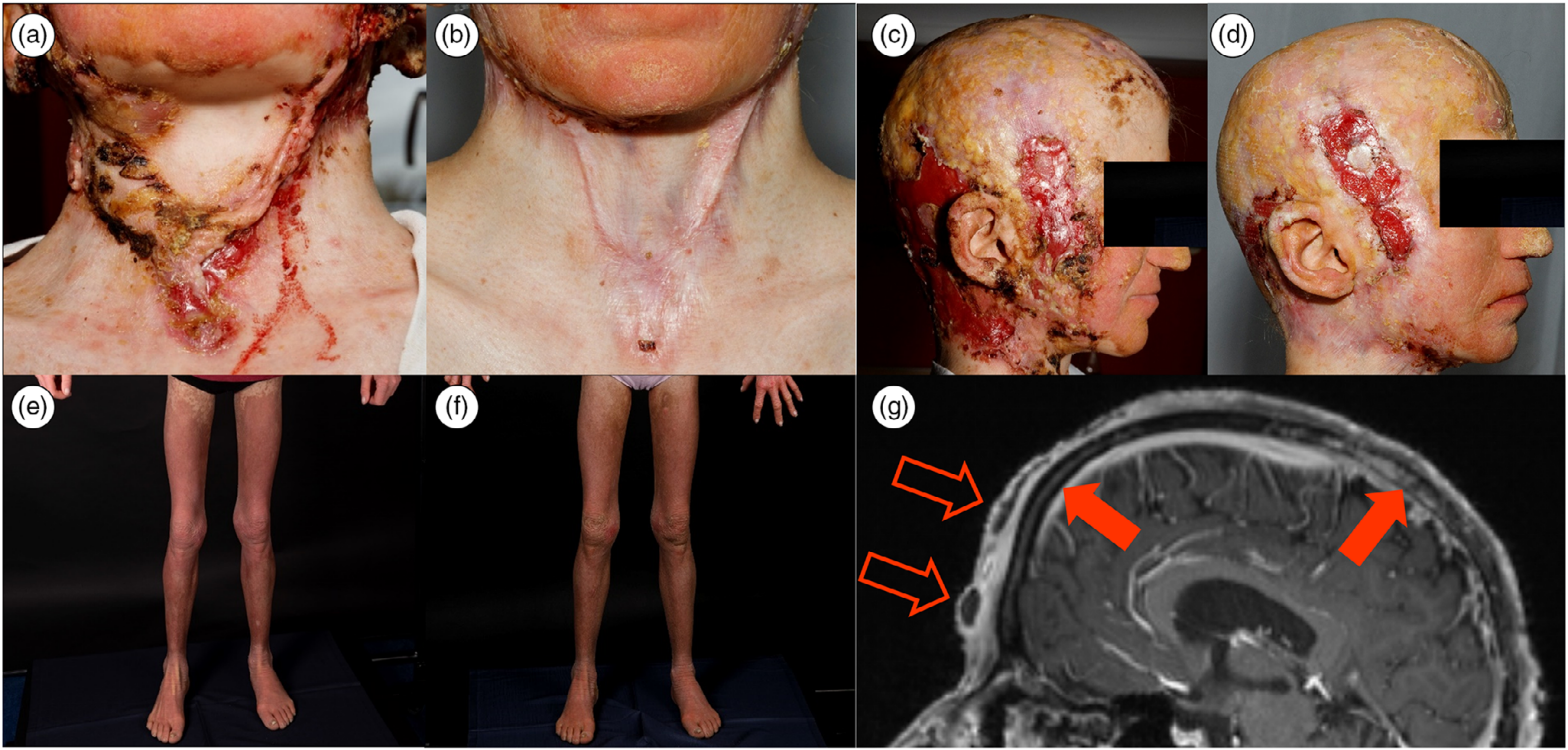
Clinical images (a, c, e) before and (b, d, f) after intervention, and (g) radiological findings. (a) Multiple hyperkeratoses, hypergranulation, and scar strands in the cervical region before surgical intervention and wound care. (b) Wound condition in the cervical region 9 months after surgical intervention. (c) Multiple hyperkeratoses, hypergranulation, and crusts in the periauricular region before surgical intervention and wound care. (d) Wound condition in the periauricular region 9 months after surgical intervention under intensive wound management; note the cicatricial alopecia. (e) Erythrokeratoderma of the legs before initiation of acitretin therapy. (f) Skin condition 6 months after initiation of acitretin therapy, showing a marked reduction in hyperkeratosis and mild decrease of erythema. (g) Sagittal T1‐weighted magnetic resonance imaging after administration of contrast medium showing increased enhancement in the frontal and parietal skull (filled arrows) corresponding to osteomyelitis as well as abscess formations (outlined arrows).

## CONFLICT OF INTEREST STATEMENT

None.
